# Lysostaphin-Coated Titan-Implants Preventing Localized Osteitis by *Staphylococcus aureus* in a Mouse Model

**DOI:** 10.1371/journal.pone.0115940

**Published:** 2014-12-23

**Authors:** Ceylan D. Windolf, Tim Lögters, Martin Scholz, Joachim Windolf, Sascha Flohé

**Affiliations:** 1 Department of Trauma- and Hand Surgery, Heinrich-Heine University Duesseldorf, Moorenstr. 5, 40225, Duesseldorf, Germany; 2 LEUKOCARE AG, Am Klopferspitz 19, 82152, Martinsried/Munich, Germany; Rockefeller University, United States of America

## Abstract

The increasing incidence of implant-associated infections induced by *Staphylococcus aureus* (SA) in combination with growing resistance to conventional antibiotics requires novel therapeutic strategies. In the current study we present the first application of the biofilm-penetrating antimicrobial peptide lysostaphin in the context of bone infections. In a standardized implant-associated bone infection model in mice beta-irradiated lysostaphin-coated titanium plates were compared with uncoated plates. Coating of the implant was established with a poly(D,L)-lactide matrix (PDLLA) comprising lysostaphin formulated in a stabilizing and protecting solution (SPS). All mice were osteotomized and infected with a defined count of SA. Fractures were fixed with lysostaphin-coated locking plates. Plates uncoated or PDLLA-coated served as controls. All mice underwent debridement and lavage on Days 7, 14, 28 to determine the bacterial load and local immune reaction. Fracture healing was quantified by conventional radiography. On Day 7 bacterial growth in the lavages of mice with lysostaphin-coated plates showed a significantly lower count to the control groups. Moreover, in the lysostaphin-coated plate groups complete fracture healing were observed on Day 28. The fracture consolidation was accompanied by a diminished local immune reaction. However, control groups developed an osteitis with lysis or destruction of the bone and an evident local immune response. The presented approach of terminally sterilized lysostaphin-coated implants appears to be a promising therapeutic approach for low grade infection or as prophylactic strategy in high risk fracture care e.g. after severe open fractures.

## Introduction

Implant-associated infections by SA are still a major challenge in trauma and orthopedic surgery even though modern operating standards and perioperative antibiotic applications minimize contamination during surgery [1]. In trauma and orthopedic surgery the presence of foreign surfaces of prosthesis or metal implants complicate the problem especially of chronified bone infections. The development of an osteomyelitis depends on both systemic host factors such as underlying diseases as diabetes, local vascularity and the degree of primary or secondary surgical tissue damage. So in general microorganisms cause an osteitis/osteomyelitis in the adult patient not alone but rather the interaction of invading microbials with an orthopedic device and the local immune response finally result in a persisting localized infection [2]. The biofilm formation of bacteria is the fundamental basis of such a chronically infected orthopedic implant [3]. Bacteria invade the host through an accidental wound or a surgical incision and attach on surfaces of implants by hydrophobic interactions [4]. There they accumulate to a multilayer cell cluster of sessile bacteria [4] and form a hydrated matrix of extracellular components including several proteins defined as biofilm [5]–[9]. This biofilm protects bacteria from the host's defenses and also dramatically increase their antibiotic resistance [1], [10]–[12]. Nevertheless, secreted protein-components of the biofilm matrix attract leukocytes and cause a local immune response. In the first line of defense, polymorphonuclear neutrophils (PMN) remain activated and secrete inflammatory factors which destroy bone and surrounding tissue [2], [13], [14] without clearing the infection. The term “frustrated phagocytosis” [15] coins the phenomenon quite well. Therefore biofilm infections are difficult to treat especially due to their inherent antibiotic resistance. Frequently, clearance of biofilm-associated infections necessitates complete implant removal. Hence, after primary attachment biofilm formation ought to be avoided [4]. In times of increasing antibiotic resistance and in the light of the failure of most antibiotic therapies alternative antimicrobial strategies becomes more and more important. One of these alternative antimicrobial substances is the antibacterial enzyme lysostaphin. Lysostaphin is a zinc ionic class III bacteriocin [16] with a molecular weight of 27 kDa [17], and contains 2 active enzymes: catalytic endopeptidase and cell wall binding domain (SH3b) [18], [19]. Lysostaphin may be able to target sessile bacteria in a biofilm and also directly destroys the extracellular biofilm matrix [20]. Pentaglycine cross bridges of cell wall components including polyglycines can be hydrolyzed by the glycylglycin endopeptidase function of lysostaphin [21]. Even methicillin-resistant SA can be rapidly eliminated avoiding undesirable systemic immune reactions [22]. The antibacterial potency of lysostaphin is well documented in animals and humans [23], [24]. Lysostaphin was applied intravenously in a rabbit model of aortic valve endocarditis [25]. The therapeutic efficacy was also demonstrated on implanted jugular vein catheters in mice [26] and in a SA induced keratitis and endophthalmitis in rabbits [27], [28]. Most SA strains are susceptible to lysostaphin due to less serine than glycine cell wall contents [21]. Although *Staphylococcus epidermidis* (SE) is known to be less sensitive to lysostaphin, biofilm produced by these bacterial strains can also be destroyed by lysostaphin when applied in higher doses [29]. In contrast lysostaphin had no visible effects on biofilms produced by *Pseudomonas aeruginosa in vitro* [29]. The systemic therapeutic application of lysostaphin may be limited by adverse side effects of the host's immune system reacting on a recombinant protein. In the context of the prevention or treatment of a localized infection at the implant/tissue interface an antimicrobial coating of implants with substances like lysostaphin seems to be a promising option. However, the prerequisite of stable and terminally sterilized bio-functionalized lysostaphin coated metal implant has so far not been addressed systematically. Therefore, the aim of this study was to evaluate an orthopedic device coated with polylactid acid in combination with stabilized lysostaphin in order to inhibit the growth of SA and to avoid implant-associated bone infections in a standardized animal model.

## Materials and Methods

### Titanium plate coating

Titanium discs grade 1 (ARA-T Advance GmbH, Dinslaken, Germany) with a size of 20×2 mm were coated with 1 mg/ml lysostaphin (ProSpec, East Brunswick, NJ, USA) per disc in a poly(D,L)-lactide (PDLLA) matrix. Lysostaphin was rebuffered in a protecting amino acid–based composition derived from the stabilizing and protecting solution platform (SPS, LEUKOCARE AG, Munich, Germany). PDLLA was dissolved in ethyl acetate (133 mg/ml) and mixed with lysostaphin to obtain a final concentration of 1 mg/ml. The loading of PDLLA on titanium plates was 17,5 µg Lysostaphin/mm^2^ and 2,33 mg PDLLA/mm^2^. The surface of the implants was 8 mm^2^ and therefore the total loading per implant was 40 µg lysostaphin and 18,64 mg PDLLA. The thickness of the lysostaphin-PDLLA coated plates using a standard coating protocol for PDLLA coatings was approximately 10 µm [30], [31], [32]. Titanium plates were cleaned with 30% (v/v) Piranha solution, a mixture of 30% hydrogen peroxide and concentrated sulfuric acid (98% H_2_SO_4_) and washed with PBS three times. Subsequently, titanium plates were dipped in the PDLLA-lysostaphin mixture (LEUKOCARE AG) and air-dried. This dip-drying step was repeated two times to obtain a multi-layer comprising PDLLA and lysostaphin. After the final drying step the coated titanium plates were sterilized with 40 kGy beta irradiation (BGS, Saale an der Donau, Germany). Terminally sterilized lysostaphin-coated titanium plates were used in microbiological screening assays and animal experiments within two weeks.

### Bacterial growth inhibition on titanium plates in vitro

The biofilm forming SA strain ATCC 29213 was cultivated in BactoTryptic Soy Broth overnight and afterwards diluted 1∶100. The coated titanium discs and uncoated control discs were incubated in the bacterial culture for 48 h at 37°C and constantly moved at 100 rpm. Bacteria were enumerated based on colony-forming units (CFU). In order to confirm the transferability into the *in vivo* osteitis mouse model titanium 4-hole MouseFix plates (RISystem, Davos, Switzerland) were coated as described before and one group of plates was additionally irradiated with 40 kGy-β for sterilization. Antibacterial activity of the implant and bacterial growth was analyzed as described above for titanium discs.

### Animals

For the experiments ten to twelve-week-old female wild-type Balb/c mice with an average weight of 21g were used (animal core facility of the Heinrich-Heine-University Duesseldorf; Germany). All animal procedures were carried out in accordance to local and national ethical guidelines and were approved by the regional ethical committee, Regional Office for Nature, Environment and Consumer Protection Nordrhein-Westfalen, Germany, with the ethical approval ID 87-51.04.2010.A112.

### Low Grade Acute Osteitis Model

A previously established implant-associated osteitis model in mice [33] was used for *in vivo* testing of coated titanium implants. Briefly, mice were anesthetized by i.p. injection of xylazine (25 mg/kg body weight) and ketamine (75 mg/kg body weight). After skin incision under sterile conditions, the fascia was opened, and the MouseFix plate was fixed to the femur. Four different treatment groups (n = 10 mice/group) were investigated: One group received titanium plates coated with 1 mg/ml lysostaphin in PDLLA per plate. One group received titanium plates coated with 1 mg/ml lysostaphin in PDLLA per plate with additional irradiation with 40 kGy-β for terminal sterilization. One control group received uncoated plates and another control group received titanium plates coated only with PDLLA. After plate fixation an osteotomy using a Gigly saw (diameter 0.22 mm, RISystem) was performed in midshaft of the femur to create a bone defect. Subsequently, 1 µl SA strain ATCC 29213 with an average CFU of 1.94E+03/µl was inoculated into the fracture gap. All mice were anesthetized again 7 and 14 days after primary operation and standardized lavage (250 µl PBS twice) with debridement of infected tissue was performed. The lavage fluid was recovered and PBS added to a total volume of 1 ml. Local debridement was performed with a surgical spoon without involving the periosteum. All surgical interventions were made by the same surgeon. At Day 28 animals were sacrificed, blood was gained by cardiac puncture and a final lavage was collected from the surgical field.

### Counts of Colony-Forming Units (CFU)

The number of colony-forming units (CFU) was elevated in the lavage on Day 7, 14, and 28. Bacteria from the thigh were gained by lavage under aseptic conditions as described before. 200 µl of lavage were serially diluted in PBS and four replicates of 10 µl of each dilution plated on Columbia agar plates with 5% sheep blood. The plates were incubated for 24 h at 37°C, and afterwards colonies were counted. Results are expressed in CFU/ml lavage fluid.

### Radiographic Analysis

On Days 0, 7, 14, and 28 after plate fixation anteroposterior radiographic images (MX20 Faxitron, Tucson, Arizona, USA) of the femora were taken. In order to rate the bone healing on radiographs we developed a score to the fracture gap size. The fracture gap size was measured at the plate opposing corticalis. The MouseFix plate has a length of 8 mm. This internal standard allows an exact calculation of the diameter of the fracture gap (Adobe Photoshop CS6). Decreasing diameters of fracture gaps representing fracture healing were rated with 1 point; a constant fracture gap meaning no healing was rated with 2 points, increasing facture gaps were rated with 3 points, obvious osteolysis with 4 and destruction of the bone were rated with 5 points.

### Analysis of Local Immune Response

The local immune response was characterized by measuring total leucocytes and PMNs in the lavage using flow cytometry (FACSCalibur BD Biosciences, Heidelberg, Germany). For neutrophilic granulocyte characterization, forward and side scatter characteristics and the myeloid cell marker APC rat anti-mouse CD11b (BD Pharmingen, Frankfurt, Germany, monoclonal, catalog number: 553991, clone: A95-1) were used.

### Quantification of IL-6 by ELISA

IL-6 levels in the lavage were analyzed (VICTOR Multilabel Counter, PerkinElmer LAS, Rodgau, Germany) using a commercially available IL-6 ELISA kit according to the manufacturer's instructions (R&D Systems, Abingdon, UK, catalog number: DY406). The lower detection limit for IL-6 was 7.5 pg/ml.

### Statistical Methods

All data are expressed as median and whiskers min to max or mean and SEM. Omnibus normality test by D'Agostino and Pearsons. Data were tested for statistical significance with two-tailed Student's t-test, Mann-Whitney-test or Wilcoxon-test using GraphPad Prism5 (GraphPad Software, San Diego, CA): *p* values ≤0.05 were considered as significant.

## Results

### Lysostaphin Retain Activity after Coating on Titan Discs

**Figure 1:**
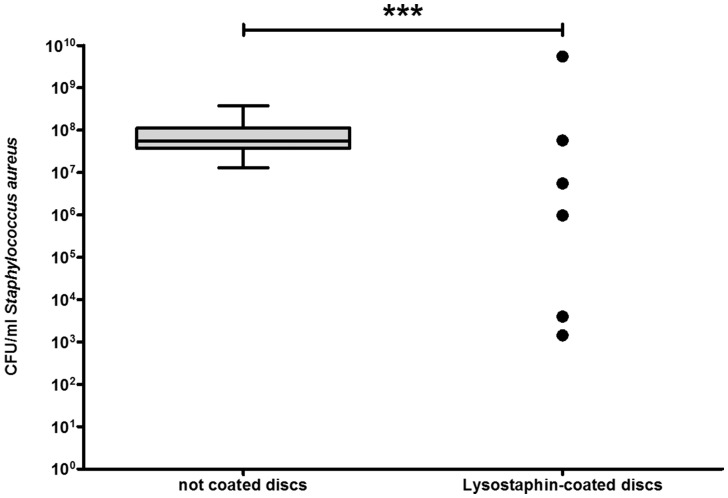
Lysostaphin-coating of titanium discs prevents bacterial growth. Titanium discs were incubated with in SA strain ATCC 29213 for 48 h. 36 of 42 cultures with lysostaphin-coated discs were sterile and thus only the six positive cultures are shown. The median CFU of cultures with uncoated discs (n = 50) was 5.62E+07 (*p*<0.0001). CFU are presented as box plots with median and whiskers min to max. Statistical analysis was performed using two-tailed Mann-Whitney-test.

**Figure 2:**
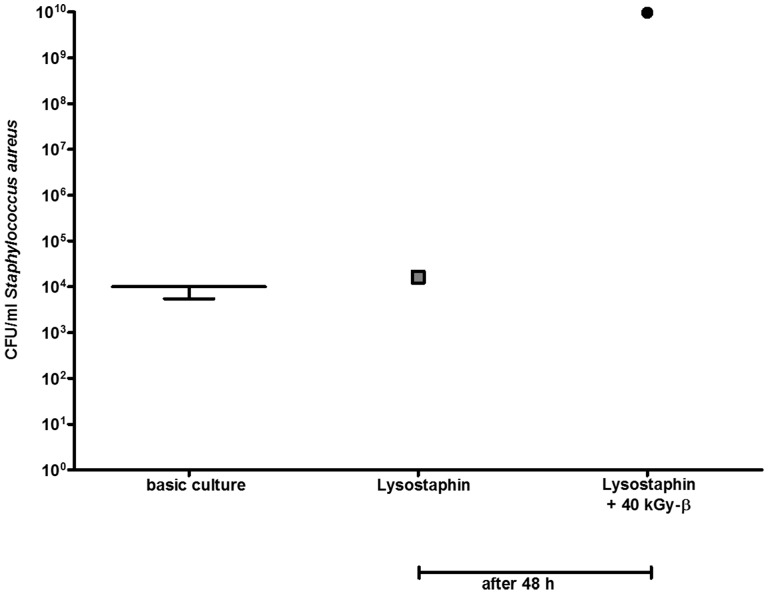
The coating method of the titan discs was transferable to the MouseFix plate. In 4 of 5 cultures of each group (MouseFix plates coated with lysostaphin and MouseFix plates coated with lysostaphin and sterilized with 40 kGy beta irradiation) no bacterial growth was measured after 48 h cultivation in a basic culture of SA with 1.00E+04 CFU/ml and thus only one positive culture of each group is shown.

First, the successful coating of lysostaphin on titanium discs was demonstrated. Bacterial culture with uncoated titanium discs revealed a median of 5.6E+07 CFU (n = 50) after 48 h incubation. In contrast, only 6 of 42 bacterial cultures with lysostaphin-coated discs showed any bacterial growth while the remaining 36 cultures were sterile after 48 h incubation (Fig. 1). Correspondingly, almost all lysostaphin-coated mouse fix plates were sterile after 48 h incubation. Only, two of 10 cultured MouseFix plates revealed bacteria growth after 48 h independent of irradiation (Fig. 2).

### Bacterial Growth and Bone Healing

**Figure 3:**
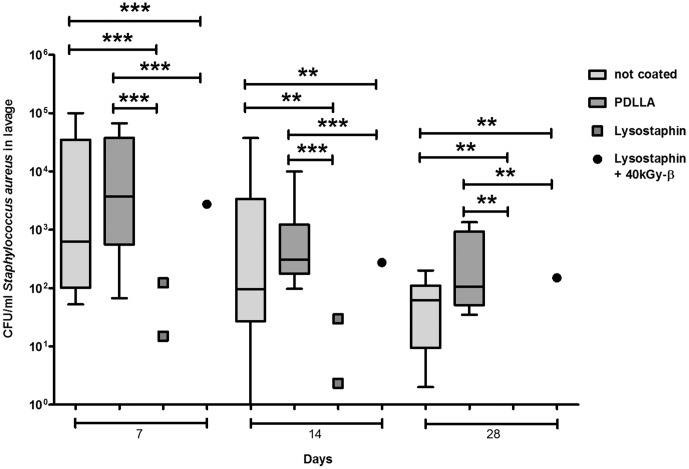
Local detection of SA around the fracture side of mice. Both groups with lysostaphin coated plates showed a significant lower bacterial count to the control groups (mice with uncoated plates, and mice with only PDLLA coated plates) at all times. In only 2 of nine respectively 1 of nine lavages in mice with lysostaphin coated plates bacterial growth was detectable with decreasing count from Day 7 to 28; all other lavages were sterile at any time. **p<0.01, ***p<0.001. CFU are presented as box plots with median and whiskers min to max. Statistical analysis was performed using two-tailed Mann-Whitney-test and Wilcoxon-test.

**Figure 4:**
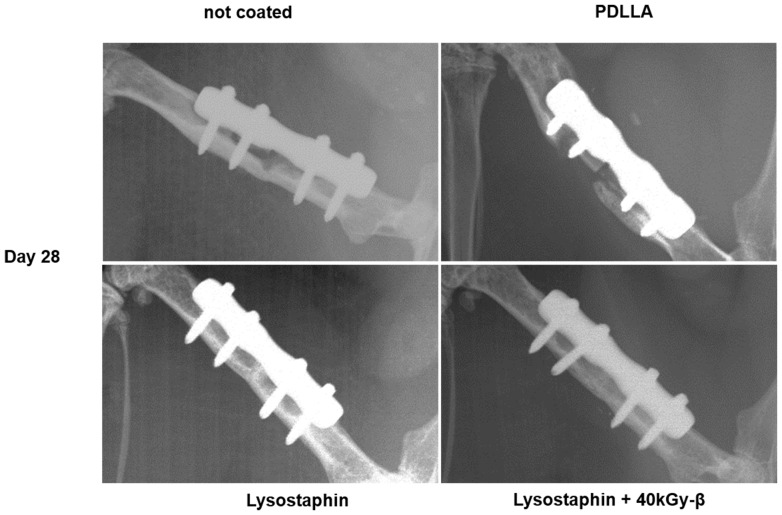
Representative x-rays of the fracture zone on Day 28. A consolidation of the fracture gap on Day 28 in both mice groups with lysostaphin-coated plates in contrast to the control groups (mice with uncoated plates, and mice with only PDLLA coated plates) after osteotomy, plate fixation and inoculation of SA with an average CFU of 1.94E+03/µl is demonstrated.

**Figure 5:**
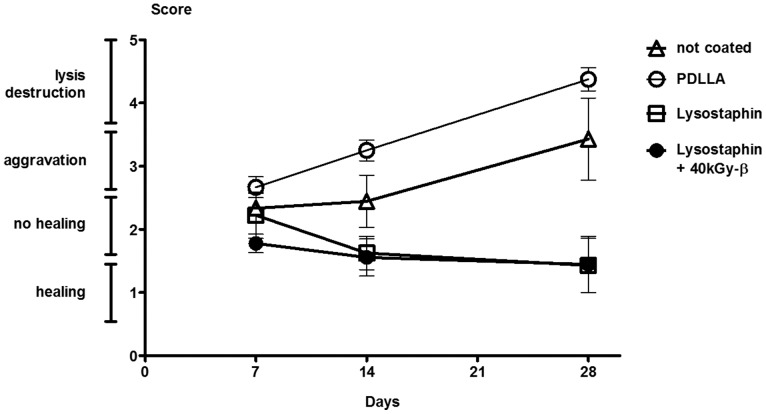
Score of fracture healing. Standardized radiographic quantification of fracture healing from mice groups with lysostaphin-coated plates (*n* = 51) vs. control groups (*n* = 50) on Days 7, 14, and 28 after osteotomy, fracture fixation and inoculation of SA. Significant differences on Day 7: not coated vs. lysostaphin +40 kGy-β (*p* = 0.0340), PDLLA vs. lysostaphin (*p* = 0.0211), PDLLA vs. lysostaphin +40 kGy-β (*p* = 0.0017). On Day 14: PDLLA vs. lysostaphin (*p* = 0.0009), PDLLA vs. lysostaphin +40 kGy-β (*p* = 0.0014). On Day 28: not coated vs. lysostaphin (*p* = 0.0186), not coated vs. lysostaphin +40 kGy-β (*p* = 0.0186), PDLLA vs. lysostaphin (*p* = 0.0014), PDLLA vs. lysostaphin +40 kGy-β (*p* = 0.0022). Radiographic quantifications are presented as mean and SEM. Statistical analysis was performed using one-tailed Mann-Whitney-test.

CFU counts in the lavage around the fracture site were measured on Days 7, 14, and 28 after primary surgery. Both the lavages of mice with uncoated plates and with the PDLLA coated control plates revealed a high and persistent bacterial load over the whole observation period of four weeks. There was no difference between mice groups with uncoated and PDLLA coated plates. However, in all mice groups with lysostaphin-coated plates bacterial growth in lavage revealed a significantly lower rate at any time point. Most lavages of the lysostaphin-coated plates were sterile (Fig. 3). Accordingly, radiographs of femora from mice with lysostaphin-coated plates showed clear signs of fracture healing by Day 14 and complete fracture consolidation by Day 28. In contrast, in all mice with uncoated or only PDLLA coated control plates fracture healing could not be observed at any time point (Fig. 4). The fracture gap was evaluated using a semi quantitative score as described above. Mice with lysostaphin-coated plates displayed a significantly higher bony healing compared to control animals. Indeed, all control groups developed clear signs of an osteitis with osteolysis or destruction of the femora (Fig. 5).

### Immune Response

**Figure 6:**
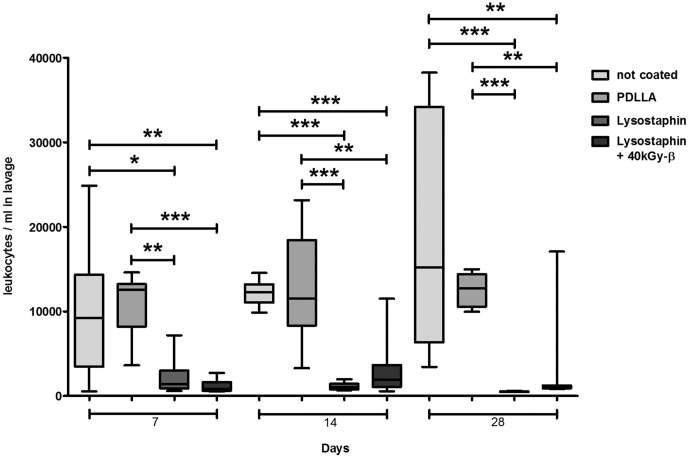
Detection of leukocytes in lavage fluid. Significantly lower counts of leukocytes in lavages of both mice groups with lysostaphin-coated plates (*n* = 52) versus control groups (mice with uncoated plates, and mice with only PDLLA coated plates; *n* = 49). *p<0.05, **p<0.01, ***p<0.001. Statistical analysis was performed using two-tailed Student's t-test and Mann-Whitney-test.

**Figure 7:**
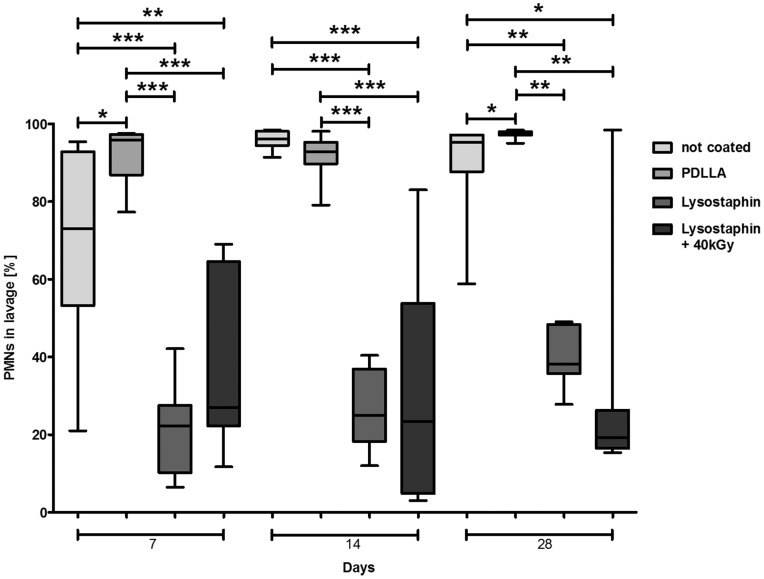
Percentage distributions of neutrophils in lavage fluid. In mice groups with lysostaphin-coated plates (*n* = 52) are less neutrophils than in the control groups (*n* = 49). *p<0.05, **p<0.01, ***p<0.001. Statistical analysis was performed using two-tailed Student's t-test and Mann-Whitney-test.

The number of leukocytes in the lavage of mice with lysostaphin-coated plates on Days 7, 14, and 28 was significantly lower than in the control groups (Fig. 6). In lysostaphin-coated implants leukocytes further decreased over time while in the uncoated control groups persistent high number of leukocytes were detectable in the lavage. In the control groups leukocytes predominantly consisted of PMNs (more than 80% at any time point). The percentage of neutrophils in the lavage was significantly lower in the lysostaphin groups (Fig. 7) at all times.

**Figure 8:**
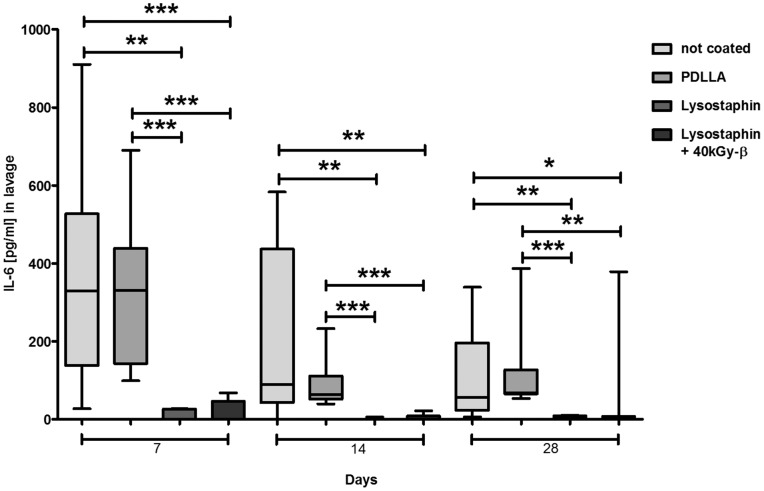
Detection of IL-6 in lavage fluids. IL-6 in lavage of both mice groups with lysostaphin-coated plates (*n* = 52) were only detectable to a minor degree in contrast to the control groups (mice with uncoated plates, and mice with only PDLLA coated plates; *n* = 49). *p<0.05, **p<0.01, ***p<0.001. Statistical analysis was performed using two-tailed Student's t-test and Mann-Whitney-test.

High numbers of IL-6 were found in the lavages on day 7 in both infected control groups without lysostaphin-coated implants which continuously decreased over time. However, also on the 28^th^ day there were still considerable numbers of IL-6 present in the lavage. In contrast IL-6 was barely detectable at any time points in all lavages of lysostaphin-coated implants (Fig. 8).

## Discussion

Osteomyelitis of implant-associated infection remains one of the major challenges in musculoskeletal surgery. Especially in severe open fractures bacterial contamination continues to be a major cause for non-union, local infection or even sepsis. Surgical debridement and antibiotic therapy are the most important treatments to control bacterial contamination. However, the efficacy of local or systemic therapy with most antibiotics in terms of eradication of surface-attached bacterial colonies with biofilm formation is very limited due to the slow growth and metabolic rate of biofilm-associated bacterial colonies [34]. Since foreign surfaces in form of metal implants are inevitably combined with fracture stabilization, biofilm-associated bacteria on the surface of orthopedic fixation devices contribute to persistent or recurrent local bone infection and delayed or missing fracture union [35]. Due to the increasing incidence of infections in orthopedic implants and growing occurrence of resistant bacteria the search for new methods to avoid implant-associated infections becomes of major importance. Novel approaches include anti-adhesive polymers, bioactive biomaterials such as chitosan derivatives or polycationic polymers and bioactive coatings with e.g. NO releasing silica nanoparticles, excellently summarized by Campoccia [36], [37]. So far most of these innovative approaches are evaluated only *in vitro* merely focusing on the bacterial growth on the foreign surface.

Based on epidemiological studies SA strains are the most frequent cause of bone infection especially in context with metal devices [6], [38], [39]. SA is known to be a potent biofilm former which further contributes to the complexity of such an infection [2]. Methicillin-resistant SA additionally limits the systemic therapeutic possibilities and therefore is a common cause for missing osseous union or even extremity amputation. This scenario calls for biofilm-penetrating substances with bactericidal activity especially against SA. The bactericidal peptide lysostaphin fulfills both prerequisites. Therefore we analyzed lysostaphin-coated metal implant in SA infection both *in vitro* and *in vivo* in a model of acute implant-associated bone infection. Surface binding of bioactive lysostaphin was accomplished with a PDLLA-carrier. PDLLA as carrier for antibiotics like gentamycin or norvancomycin [40]–[42] or pharmaceuticals like zoledronic acid [43] or growth factors like TGF-β [44] *in vitro* is well established. PDLLA is biocompatible, durable on implants, and does not induce any undesirable immune reactions [45]. However, PDLLA-dependent coating of lysostaphin has not been described before.

The two stereoisomers of lactic acid, L-lactic acid and D-lactic acid can produce four distinct materials: Poly(*D*-lactic acid) (PDLA), a crystalline material with a regular chain structure; poly(*L*-lactic acid) (PLLA), which is hemicrystalline, and likewise with a regular chain structure; poly(*D*,*L*-lactic acid) (PDLLA) which is highly hydrophobic and amorphous; and *meso*-PLA, obtained by the polymerization of *meso*-lactide. PDLA, PLLA and PDLLA are soluble in common solvents including ethyl acetate, benzene, chloroform, dioxane, etc. and degrade by simple hydrolysis of the ester bond even in the absence of a hydrolase [46], [47]. In this work the coating is based on an amorphous polymer of low molecular weight poly(D,L-lactic acid), Resomer R203H with molecular weights M_w_ between 18000–24000 Da. Surprisingly, SPS-formulated lysostaphin remained stable and bioactive after PDLLA coating even after terminal sterilization of the coated implants by 40 kGy beta-radiation. From the regulatory point of view, terminal sterilization is required for the approval of medical devices whenever it is achievable. Moreover, increased stability of biomolecule-coated implants may prevent storage-related loss of function. Embedding the SPS-stabilized antibacterial enzyme lysostaphin in the dried amorphous matrix of poly(D,L-lactide) might be an interesting approach to clinically address the formation of SA-related biofilms on implants in the future.

In this work we demonstrated for the first time the successful coating of lysostaphin on titan implants and its effectiveness in an *in vivo* mouse model. Bacterial growth of the contaminated fracture gap was almost completely avoided. Consecutively, osseous consolidation was achieved with lysostaphin-coated implants in spite of the initial bacterial contamination. In addition, infection-associated local inflammation was also prevented by the application of a lysostaphin-coated implant. At least in this mouse model of acute bone infection lysostaphin-coated implants seem to be a very promising therapeutic approach.

The potency of lysostaphin has been described in several local applications, i.e. as cream to reduce nasal colonization in rat [48]. Intravenous application of lysostaphin can reduce systemic- and organ infections in mice [49]. Lysostaphin disrupts biofilm *in vitro* and additionally acts bactericidal to SA strains and with a smaller efficacy also to SE [20]. Nevertheless, lysostaphin will not be the ultimate miracle cure for all musculoskeletal infections. Until now, only few lysostaphin-resistant SA strains have been reported but mutations to a higher amount of serine in the peptidoglycan of SA cell wall will result in diminishing anti-staphylococcal effect of this substance [21]. In addition, antibacterial efficacy of lysostaphin is limited to SA and in higher concentration to SE. So far only metal implants coated with agents like vancomycin or gentamycin are available. However, both of these antibiotics do not penetrate biofilm [50], [51]. In the context of the biofilm-associated infection coatings with vancomycin or gentamycin do not seem to be the perfect choice of antibiotic substance. But a combination of the glycopeptide antibiotics vancomycin or gentamycin with lysostaphin would be a potential weapon against SA even after biofilm formation [25].

Of course the lysostaphin-coated implants as described in this experimental series have some limitations. Firstly, we were not able to achieve a sterile culture in all samples *in vitro* as an evidence for some inconsistency in the drug-eluting efficacy of the PDLLA-coating procedure. Secondly, long term stability of the PDLLA-lysostaphin-coating needs to be evaluated. Further, in the experimental set up lysostaphin-coated implants were present with the onset of the infection. Therefore, the presented data actually do not allow any conclusions concerning the efficacy of lysostaphin in an established bone infection. Based on the presented *in vivo* data however, we can demonstrate that lysostaphin-coating completely prevents the development of an osteomyelitis in spite of a significant bacterial contamination and the presence of a metal surface, normally leading to persisting infections due to biofilm formation. From a clinical point of view such a strategy is quite realistic. Antimicrobial coating of orthopedic fixation devices would be desirable in open fracture treatment or in high risk osteosynthesis e.g. in elderly patients with an impaired immune response. In addition, after the infection has been clinically resolved such an antimicrobial coating of an implant would be of special use in septic bone surgery to ensure a minimized risk of reinfection after secondary internal fixation. In such situations an antimicrobial coating of the implant could be of interesting choice to avoid recurrence of infection. The same holds true for the emerging problem of septic revision arthroplasty.

## Conclusion

Biofilm-associated bone infections prevent osseous consolidation and are a significant problem e.g. in the treatment of open fractures. Lysostaphin, as a potential therapeutic agent coated on implant surfaces with a common carrier like PDLLA avoids an osteitis in mice femur and results in a complete consolidation of a fracture gap in spite of inoculation with SA.

Therefore, lysostaphin-coated fixation devices are a promising therapeutic strategy for open fractures or septic revision surgery.
